# Supplementary material for: Bioinformatics Prediction for Network-Based Integrative Multi-Omics Expression Data Analysis in Hirschsprung Disease
Source: Biomolecules. 2024 Jan 30;14(2):164. doi: 10.3390/biom14020164 (PMC10886964; doi:10.3390/biom14020164)
Supplement: Supplementary file 1 [file biomolecules-14-00164-s001.zip › biomolecules-2784092-supplementary/Supplementary_files/Figure S3.pdf]

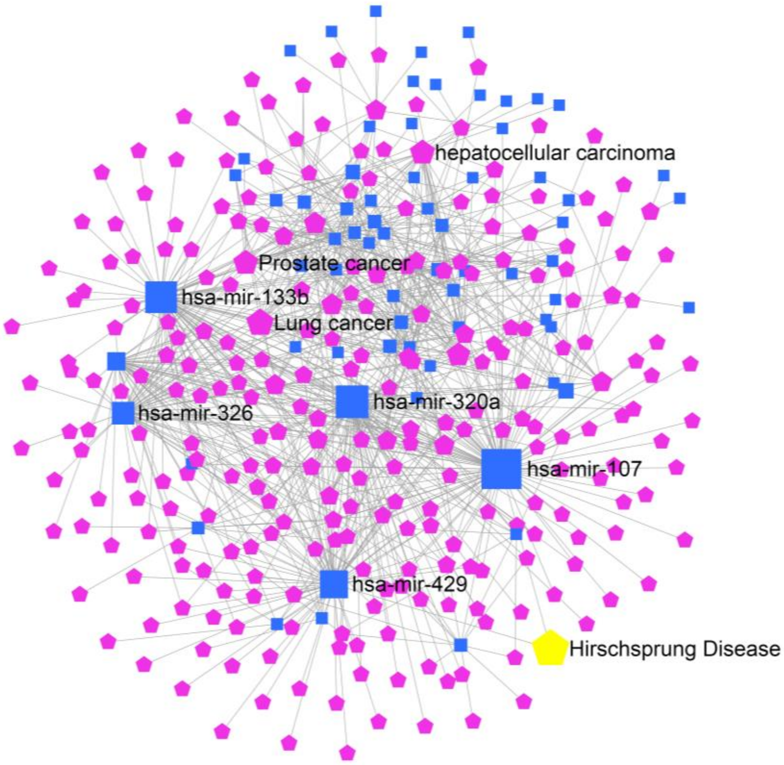

Figure S3. MiRNA-Disease network. Polygon nodes in pink correspond to diseases and the rectangular nodes in blue are miRNAs. The sizes of the nodes are proportional to their respective degrees. Hirschsprung disease is highlighted in yellow.
