# Supplementary material for: Bioinformatics Prediction for Network-Based Integrative Multi-Omics Expression Data Analysis in Hirschsprung Disease
Source: Biomolecules. 2024 Jan 30;14(2):164. doi: 10.3390/biom14020164 (PMC10886964; doi:10.3390/biom14020164)
Supplement: Supplementary file 1 [file biomolecules-14-00164-s001.zip › biomolecules-2784092-supplementary/Supplementary_files/Table S1.pdf]

Supplementary Table S1. Characteristics of the selected datasets for this study.

DataSet	Repository	Instrument	Omic expression Designs	Type of omics expression data	Total number of samples (n)	Original Publications
GSE98502	Gene Expression Omnibus (GEO)	Agilent-071342 LncRNA	Single sample design	mRNA	16	[28]
GSE96854	Gene Expression Omnibus (GEO)	NimbleGen Human Gene Expression 12x135K Array	Pooling sample design	mRNA	6	[29]
GSE77296	Gene Expression Omnibus (GEO)	Exiqon miRCURY LNA microRNA array, 7th generation	Single sample design	miRNA	12	[30]
PXD021292	Integrated Proteome resources (iProX)	Q Exactive HF	Pooling sample design	Protein	6	[31]

miRNA, microRNA
