# Supplementary material for: Bioinformatics Prediction for Network-Based Integrative Multi-Omics Expression Data Analysis in Hirschsprung Disease
Source: Biomolecules. 2024 Jan 30;14(2):164. doi: 10.3390/biom14020164 (PMC10886964; doi:10.3390/biom14020164)
Supplement: Supplementary file 1 [file biomolecules-14-00164-s001.zip › biomolecules-2784092-supplementary/Supplementary_files/Table S2.pdf]

Supplementary Table S2. Terms enriched in all three datasets comparing stenotic colon to ganglionic colon samples.

Category	Identifier	Description
Biological Process	GO:0061564	Axon development
	GO:0007155	Cell adhesion
	GO:0048666	Neuron development
	GO:0048812	Neuron projection morphogenesis
	GO:0007409	Axonogenesis
	GO:0048667	Cell morphogenesis involved in neuron differentiation
	GO:0007610	Behavior
	GO:0032989	Cellular component morphogenesis
	GO:0031175	Neuron projection development
	GO:0000904	Cell morphogenesis involved in differentiation
Cellular Component	GO:0043005	Neuron projection
	GO:0030424	Axon
	GO:0045202	Synapse
	GO:0036477	Somatodendritic compartment
	GO:0098685	Schaffer collateral - ca1 synapse
	GO:0098793	Presynapse
Disease	C1510586	Autism spectrum disorders
	C0206754	Neuroendocrine tumors
	C1535926	Neurodevelopmental disorders
	C0030193	Pain
Drug	CID000036511	Substance P
	6173_UP	2-propylpentanoic acid; Up 200; 200uM; HL60; HT_HG-U133A
	CID000004624	6-OHDA
	ctd:D008704	Methazolamide
	3604_UP	Demeclocycline hydrochloride [64-73-3]; Up 200; 8uM; MCF7; HT_HG-U133A
	CID000005202	Serotonin
	CID000128644	Lant-6
	CID000004376	N-methyl-DL-aspartic acid
	CID000000681	Dopamine
	CID000000813	Potassium
	CID000042785	Lupex
	ctd:D003561	Cytarabine
	CID000005775	Phentolamine
	ctd:D015122	6-Mercaptopurine
	ctd:D010278	Parathion
	CID000004425	EN-1530
	CID000013296	4-ethylsulfonylnaphthalene-1-sulfonamide
	ctd:C015001	Arsenite
	CID000446537	C-cluster
	CID000002551	Carbachol
	5615_UP	Cephalexin monohydrate [23325-78-2]; Up 200; 11uM; MCF7; HT_HG-U133A
	CID000161645	Oriens
	CID000000119	Gamma-aminobutyric acid
	3520_UP	Moricizine hydrochloride [31883-05-3]; Up 200; 8.6uM; MCF7; HT_HG-U133A
	CID000002833	Colchine
	CID000000611	Glutamate
	CID000000187	Acetylcholine
	3639_UP	3-alpha-Hydroxy-5-beta-androstan-17-one [53-42-9]; Up 200; 13.8uM; MCF7; HT_HG-U133A
	CID000000951	Noradrealine
