# Supplementary material for: Bioinformatics Prediction for Network-Based Integrative Multi-Omics Expression Data Analysis in Hirschsprung Disease
Source: Biomolecules. 2024 Jan 30;14(2):164. doi: 10.3390/biom14020164 (PMC10886964; doi:10.3390/biom14020164)
Supplement: Supplementary file 1 [file biomolecules-14-00164-s001.zip › biomolecules-2784092-supplementary/Supplementary_files/Table S3.pdf]

Supplementary Table S3. List of Potential Disease associated modules

Disease associated module	Cluster size	Adjusted p-value	List of Genes
1	21	0.02	*SLC6A15, SLC2A1[1], RHOV, RHOA, RAC3, RAC2, NRAS, MARCKSL1, KRAS[1], JPH1, FLOT1, FERMT2, CSE1L, CPNE8, CDC42BPB, CDC42, CAV1[1], BCCIP, ATP6V1H, ATP2B1, AKAP12*
2	10	0.04	*RAC2, NOTCH2, NOTCH1, MARCKSL1, LIMA1, KRAS[1], JAG1, FAF2, CFTR, CAV1[1]*
3	9	0.04	*ZC3H18, SLC25A6, RET[1], PRPF8, PKM, MAPK3[1], FBXO7, EFTUD2, BAG6*
4	9	0.04	*PTPN6, PTPN11, PTPN1, PLCG1, MAPK3[1], KDR, IRS1, INSR, CAV1[1]*
5	8	0.04	*SRC, PPP1R9A, PPP1CA, NRAS, MAPK3[1], LRP8, HDAC6, CAV1[1]*
6	8	0.04	*YWHAE, YAP1, SLC9A3R2[1], PTPRF, PTPN14, MAP2K1, KRAS[1], CTNNB1*
7	7	0.02	*TBC1D10A, SLC9A3R2[1], RPS6KA1, PODXL, KRAS[1], CTNNB1, CTNNA1*
8	5	0.02	*RASA1, PLCG1, NRG1[1], ERBB2, CAV1[1]*
9	5	0.02	*TRIP10, MAPK3[1], HTT, HSF1, CBS[1]*
10	5	0.02	*TULP3, SFN, MAPK3[1], KRAS[1], BRAF*
11	5	0.02	*TFRC, ROBO2, PPP1R1B, KRAS[1], CAV1[1]*
12	5	0.02	*PLCG1, PICALM, KRAS[1], GAB1, CAV1[1]*
13	5	0.02	*SLC2A1[1], MYC, MAPK3[1], ILK, ATP2A2*
14	5	0.02	*SUMO2, SLC2A1[1], RHOB, CAV1[1], ARHGAP35*
15	5	0.02	*TXNL4A, RET[1], KRAS[1], CTNND1, CDH2*
16	5	0.02	*KRAS[1], DTNA, DMD, DAG1, CAV1[1]*
17	5	0.02	*SNAP23, KRAS[1], EMD, CFTR, BANF1[1]*
18	5	0.02	*PTPRJ, MAPK3[1], KRAS[1], FAM83D, DYNC1I1*
19	5	0.02	*KRAS[1], FRS2, FLOT2, FLOT1, CAV1[1]*
20	5	0.02	*SYNCRIP, SNRNP70[1], RET[1], HNRNPU, H3C1, H3C4, H3C3, H3C6, H3C11, H3C8, H3C12, H3C10, H3C2, H3C7*
21	5	0.02	*TULP3, PTPN6, MAPK3[1], LYN, KRAS[1]*
22	5	0.02	*TMEM87A, SYP[1], NRG1[1], MBOAT7, KRAS[1]*
23	5	0.02	*SCARB1, NDUFA12, MMGT1, KRAS[1], CAV1[1]*
24	5	0.02	*RAB1A, KRAS[1], GORASP2, GOLGA2, ENO2[1]*

Supplementary Table S3. (Continued)

Disease associated module	Cluster ID	Cluster size	Adjusted p-value	List of Genes
25	537	5	0.02	*XRCC3, VCAM1, UBR4*[1]*, SNRNP70*[1]*, SLC2A1*[1]
26	541	5	0.02	*UBR4*[1]*, SERPINH1, HTR2C, CAV1*[1]*, AGTR1*
27	545	5	0.02	*SLC9A3R2*[1]*, SCRIB, NOX1, KRAS*[1]*, ARHGEF7*
28	551	5	0.02	*PGD, KRAS*[1]*, CAV1*[1]*, BSG, APP*
29	570	5	0.02	*SLC19A1, KRAS*[1]*, GJA1, CAV1*[1]*, ACTB*
30	588	5	0.02	*SLC9A3R2*[1]*, PPT1, EZR, ENO2*[1]*, CUL2*
31	593	5	0.02	*STMN2*[1]*, MAPT, MAPK3*[1]*, FKBP5, ARRB2*
32	596	5	0.02	*TMTC4, SYP*[1]*, SLC25A16, NRG1*[1]*, B3GNT2*
33	597	5	0.02	*SNRNP70*[1]*, MAPK3*[1]*, LUC7L, GEMIN5, C1QBP*
34	630	5	0.02	*NRG1*[1]*, LSR, LEMD3, KRAS*[1]*, CDC5L*
35	631	5	0.02	*MARVELD2, KRAS*[1]*, GJB7, GAP43*[1]*, APP*
36	635	5	0.02	*SNRNP70*[1]*, METTL14, H3C1, H3C4, H3C3, H3C6, H3C11, H3C8, H3C12, H3C10, H3C2, H3C7, BMI1, BANF1*[1]
37	638	5	0.02	*TFRC, KRAS*[1]*, CAV1*[1]*, ALDH1A3, ABCC1*
38	662	5	0.02	*XRCC3, UBR4*[1]*, SNRNP70*[1]*, PRPF40A, RBMXL1*
39	671	5	0.02	*SLC9A3R2*[1]*, PTEN, ESR1, CAV1*[1]*, BMX*
40	697	5	0.02	*TMEM87A, SYP*[1]*, SLC20A2, KRAS*[1]*, ADCY6*
41	698	5	0.02	*TMEM164, SYP*[1]*, SLC35B2, NRG1*[1]*, CISD2*
42	700	5	0.02	*SYP*[1]*, SLC25A23, NRG1*[1]*, MTCH1, CNTNAP3*
43	710	5	0.02	*SOX4, KRAS*[1]*, EMD, DDHD1, BANF1*[1]
44	713	5	0.02	*SURF4, STOM, SLC2A1*[1]*, KRAS*[1]*, CCR2*
45	751	5	0.02	*SNRNP70*[1]*, ENO2*[1]*, CUL4A, CUL2, CUL1*
46	848	4	0.02	*HSPE1, GAL*[1]*, ENOPH1, CBS*[1]
47	959	4	0.02	*LNPK, CHMP7, CCK*[1]*, CAV1*[1]
48	1001	4	0.02	*KIF1A, ESR1, CAV1*[1]*, BANF1*[1]

Supplementary Table S3. (Continued)

**Disease associated module**	**Cluster ID**	**Cluster size**	**Adjusted p-value**	**List of Genes**
49	1093	4	0.02	*UBR4*[1], *SLC9A3R2*[1], *PRKD1, C1QBP*
50	1107	4	0.02	*UBE2I, SLC2A1*[1], *CBS*[1], *BLMH*
51	1191	4	0.02	*SLC9A3R2*[1], *KIFAP3, GAP43*[1], *CYP2S1*
52	1218	4	0.02	*UBR4*[1], *PI4KA, KRAS*[1], *ADAM9*
53	1244	4	0.02	*SREBF2, NRG1*[1], *MAPK3*[1], *CSTF2T*
54	1254	4	0.02	*THRAP3, SNRNP70*[1], *CAV1*[1], *CAPN1*
55	1643	4	0.02	*MMP10, GAL*[1], *CBS*[1]

[1]HDEGs in cluster
