# Supplementary material for: Bioinformatics Prediction for Network-Based Integrative Multi-Omics Expression Data Analysis in Hirschsprung Disease
Source: Biomolecules. 2024 Jan 30;14(2):164. doi: 10.3390/biom14020164 (PMC10886964; doi:10.3390/biom14020164)
Supplement: Supplementary file 1 [file biomolecules-14-00164-s001.zip › biomolecules-2784092-supplementary/Supplementary_files/Table S4.pdf]

Supplementary Table S4. List of predicted Hirschsprung-related genes.

Gene symbol	Approved name	HGNC ID	Significant score	Nº significant cluster present	Interactor type	Gene type	Evidence of gene-disease relationship	Evidence of gene-ENS relationship
ABCC1	ATP binding cassette subfamily C member 1	HGNC:51	3	1	OG		None	None
ACTB	Actin beta	HGNC:132	3	1	OG	Essential	DisGeNET and Literature	None
ADAM9	ADAM metallopeptidase domain 9	HGNC:216	3	1	OG	Oncogen	None	None
ADCY6	Adenylate cyclase 6	HGNC:237	3	1	OG		None	None
AGTR1	Angiotensin II receptor type 1	HGNC:336	3	1	OG	Tumor suppressor gene	None	None
AKAP12	A-kinase anchoring protein 12	HGNC:370	3	1	ODEG	Tumor suppressor gene	None	None
ALDH1A3	Aldehyde dehydrogenase 1 family member A3	HGNC:409	3	1	OG		None	None
APP	Amyloid beta precursor protein	HGNC:620	3	2	OG		DisGeNET and Literature	Literature
ARHGAP35	Rho GTPase activating protein 35	HGNC:4591	3	1	ODEG	House-keeping gene	Literature	None
ARHGEF7	Rho guanine nucleotide exchange factor 7	HGNC:15607	3	1	ODEG		None	None
ARRB2	Arrestin beta 2	HGNC:712	3	1	OG		None	None
ATP2A2	ATPase sarcoplasmic/endoplasmic reticulum Ca2+ transporting 2	HGNC:812	3	1	ODEG	Essential	None	None
ATP2B1	ATPase plasma membrane Ca2+ transporting 1	HGNC:814	3	1	ODEG		None	None
ATP6V1H	ATPase H+ transporting V1 subunit H	HGNC:18303	3	1	ODEG		None	None
B3GNT2	UDP-GlcNAc:betaGal beta-1,3-N-acetylglucosaminyltransferase 2	HGNC:15629	3	1	OG		None	None
BAG6	BAG cochaperone 6	HGNC:13919	2.7	1	OG	House-keeping gene	None	Literature
BCCIP	BRCA2 and CDKN1A interacting protein	HGNC:978	3	1	ODEG	House-keeping gene, Essential	None	None
BLMH	Bleomycin hydrolase	HGNC:1059	3	1	ODEG		None	None
BMI1	BMI1 proto-oncogene, polycomb ring finger	HGNC:1066	3	1	OG	House-keeping gene, Oncogen, Transcription factor	CTD, DisGeNET and Literature	None
BMX	BMX non-receptor tyrosine kinase	HGNC:1079	3	1	OG		None	None
BRAF	B-Raf proto-oncogene, serine/threonine kinase	HGNC:1097	3	1	OG	Oncogen, Essential	Literature	None
BSG	Basigin (Ok blood group)	HGNC:1116	3	1	OG	House-keeping gene	None	None
C1QBP	Complement C1q binding protein	HGNC:1243	3	2	ODEG	Essential	None	Literature
CAPN1	Calpain 1	HGNC:1476	3	1	OG		DisGeNET and Literature	None

Supplementary Table S4. (Continued)

Gene symbol	Approved name	HGNC ID	Significant score	Nº significant cluster present	Interactor type	Gene type	Evidence of gene-disease relationship	Evidence of gene-ENS relationship
CCR2	C-C motif chemokine receptor 2	HGNC:1603	3	1	OG		Literature	Literature
CDC42	Cell division cycle 42	HGNC:1736	3	1	ODEG	House-keeping gene	Literature	Literature
CDC42BPB	CDC42 binding protein kinase beta	HGNC:1738	3	1	ODEG	House-keeping gene	None	None
CDC5L	Cell division cycle 5 like	HGNC:1743	3	1	OG	House-keeping gene, Oncogen, Essential, Transcription factor	None	None
CDH2	Cadherin 2	HGNC:1759	3	1	ODEG		Malacards	Literature
CFTR	CF transmembrane conductance regulator	HGNC:1884	2.7	2	ODEG	Tumor suppressor gene	None	Literature
CHMP7	Charged multivesicular body protein 7	HGNC:28439	3	1	ODEG		None	None
CISD2	CDGSH iron sulfur domain 2	HGNC:24212	3	1	OG		None	None
CNTNAP3	Contactin associated protein family member 3	HGNC:13834	3	1	OG		None	None
CPNE8	Copine 8	HGNC:23498	3	1	ODEG		None	None
CSE1L	Chromosome segregation 1 like	HGNC:2431	3	1	ODEG	Essential	None	None
CSTF2T	Cleavage stimulation factor subunit 2 tau variant	HGNC:17086	3	1	OG		None	None
CTNNA1	Catenin alpha 1	HGNC:2509	3	1	OG		None	None
CTNNB1	Catenin beta 1	HGNC:2514	2.7	2	OG	House-keeping gene, Oncogen, Transcription factor	Literature	Literature
CTNND1	Catenin delta 1	HGNC:2515	3	1	OG	Tumor suppressor gene	None	None
CUL1	Cullin 1	HGNC:2551	3	1	OG	House-keeping gene, Tumor suppressor gene, Essential	None	None
CUL2	Cullin 2	HGNC:2552	3	2	OG	Tumor suppressor gene	None	None
CUL4A	Cullin 4A	HGNC:2554	3	1	OG	Oncogen	None	None
CYP2S1	Cytochrome P450 family 2 subfamily S member 1	HGNC:15654	3	1	OG		None	None
DAG1	Dystroglycan 1	HGNC:2666	3	1	OG	House-keeping gene	None	None
DDHD1	DDHD domain containing 1	HGNC:19714	3	1	OG		None	None
DMD	Dystrophin	HGNC:2928	3	1	ODEG	Tumor suppressor gene	None	Literature

Supplementary Table S4. (Continued)

Gene symbol	Approved name	HGNC ID	Significant score	Nº significant cluster present	Interactor type	Gene type	Evidence of gene-disease relationship	Evidence of gene-ENS relationship
DTNA	Dystrobrevin alpha	HGNC:3057	3	1	OG		None	None
DYNC1I1	Dynein cytoplasmic 1 intermediate chain 1	HGNC:2963	3	1	ODEG		None	None
EFTUD2	Elongation factor Tu GTP binding domain containing 2	HGNC:30858	2.7	1	ODEG	Essential	None	None
EMD	Emerin	HGNC:3331	3	2	OG	House-keeping gene	None	None
ENOPH1	Enolase-phosphatase 1	HGNC:24599	3	1	ODEG		None	None
ERBB2	Erb-b2 receptor tyrosine kinase 2	HGNC:3430	3	1	OG	Oncogen	DisGeNET, HuGENet, Malacards and Literature	Literature
ESR1	Estrogen receptor 1	HGNC:3467	3	2	OG	Tumor suppressor gene, Transcription factor	DisGeNET and Literature	None
EZR	Ezrin	HGNC:12691	3	1	OG		None	None
FAF2	Fas associated factor family member 2	HGNC:24666	2.7	1	OG	House-keeping gene	None	None
FAM83D	Family with sequence similarity 83 member D	HGNC:16122	3	1	OG	Oncogen	None	None
FBXO7	F-box protein 7	HGNC:13586	2.7	1	ODEG		None	None
FERMT2	FERM domain containing kindlin 2	HGNC:15767	3	1	ODEG		None	None
FKBP5	FKBP prolyl isomerase 5	HGNC:3721	3	1	OG		None	None
FLOT1	Flotillin 1	HGNC:3757	3	2	ODEG		None	None
FLOT2	Flotillin 2	HGNC:3758	3	1	OG	House-keeping gene	None	None
FRS2	Fibroblast growth factor receptor substrate 2	HGNC:16971	3	1	OG		Literature	Literature
GAB1	GRB2 associated binding protein 1	HGNC:4066	3	1	OG		None	None
GEMIN5	Gem nuclear organelle associated protein 5	HGNC:20043	3	1	OG	Essential	None	None
GJA1	Gap junction protein alpha 1	HGNC:4274	3	1	OG	Tumor suppressor gene	Literature	Literature
GJB7	Gap junction protein beta 7	HGNC:16690	3	1	OG		None	None
GOLGA2	Golgin A2	HGNC:4425	3	1	ODEG	House-keeping gene	None	None
GORASP2	Golgi reassembly stacking protein 2	HGNC:17500	3	1	OG	House-keeping gene	None	None
H3C1	H3 clustered histone 1	HGNC:4766	3	2	OG		None	None
H3C10	H3 clustered histone 10	HGNC:4775	3	2	OG		None	None
H3C11	H3 clustered histone 11	HGNC:4771	3	2	OG		None	None

Supplementary Table S4. (Continued)

Gene symbol	Approved name	HGNC ID	Significant score	Nº significant cluster present	Interactor type	Gene type	Evidence of gene-disease relationship	Evidence of gene-ENS relationship
H3C12	H3 clustered histone 12	HGNC:4774	3	2	OG		None	None
H3C2	H3 clustered histone 2	HGNC:4776	3	2	OG		None	None
H3C3	H3 clustered histone 3	HGNC:4768	3	2	OG		None	None
H3C4	H3 clustered histone 4	HGNC:4767	3	2	OG		None	None
H3C6	H3 clustered histone 6	HGNC:4769	3	2	OG		None	None
H3C7	H3 clustered histone 7	HGNC:4773	3	2	OG		None	None
H3C8	H3 clustered histone 8	HGNC:4772	3	2	OG		None	None
HDAC6	Histone deacetylase 6	HGNC:14064	2.7	1	OG		None	Literature
HNRNPU	Heterogeneous nuclear ribonucleoprotein U	HGNC:5048	3	1	ODEG	House-keeping gene, Essential	None	None
HSF1	Heat shock transcription factor 1	HGNC:5224	3	1	OG	House-keeping gene, Transcription factor	None	None
HSPE1	Heat shock protein family E (Hsp10) member 1	HGNC:5269	3	1	ODEG	House-keeping gene, Essential	None	None
HTR2C	5-hydroxytryptamine receptor 2C	HGNC:5295	3	1	OG		None	None
HTT	Huntingtin	HGNC:4851	3	1	OG	House-keeping gene	None	Literature
ILK	Integrin linked kinase	HGNC:6040	3	1	OG	Tumor suppressor gene, Oncogen	None	None
INSR	Insulin receptor	HGNC:6091	2.7	1	OG		None	None
IRS1	Insulin receptor substrate 1	HGNC:6125	2.7	1	ODEG		Literature	Literature
JAG1	Jagged canonical Notch ligand 1	HGNC:6188	2.7	1	ODEG		None	None
JPH1	Junctophilin 1	HGNC:14201	3	1	ODEG		None	None
KDR	Kinase insert domain receptor	HGNC:6307	2.7	1	OG		Malacards	Literature
KIF1A	Kinesin family member 1A	HGNC:888	3	1	ODEG		Literature	None
KIFAP3	Kinesin associated protein 3	HGNC:17060	3	1	OG		None	None
LEMD3	LEM domain containing 3	HGNC:28887	3	1	OG		None	None
LIMA1	LIM domain and actin binding 1	HGNC:24636	2.7	1	OG	Tumor suppressor gene	None	None
LNPK	Lunapark, ER junction formation factor	HGNC:21610	3	1	ODEG		None	None
LRP8	LDL receptor related protein 8	HGNC:6700	2.7	1	ODEG		None	None

Supplementary Table S4. (Continued)

Gene symbol	Approved name	HGNC ID	Significant score	Nº significant cluster present	Interactor type	Gene type	Evidence of gene-disease relationship	Evidence of gene-ENS relationship
LSR	Lipolysis stimulated lipoprotein receptor	HGNC:29572	3	1	OG		None	None
LUC7L	LUC7 like	HGNC:6723	3	1	OG		None	None
LYN	LYN proto-oncogene, Src family tyrosine kinase	HGNC:6735	3	1	OG		None	None
MAP2K1	Mitogen-activated protein kinase kinase 1	HGNC:6840	2.7	1	OG	House-keeping gene	None	None
MAPT	Microtubule associated protein tau	HGNC:6893	3	1	ODEG		Malacards and Literature	Literature
MARCKSL1	MARCKS like 1	HGNC:7142	2.7	2	ODEG		None	None
MARVELD2	MARVEL domain containing 2	HGNC:26401	3	1	OG		None	None
MBOAT7	Membrane bound O-acyltransferase domain containing 7	HGNC:15505	3	1	OG		None	None
METTL14	Methyltransferase 14, N6-adenosine-methyltransferase subunit	HGNC:29330	3	1	OG	Essential	None	None
MMGT1	Membrane magnesium transporter 1	HGNC:28100	3	1	OG	Essential	None	None
MMP10	Matrix metallopeptidase 10	HGNC:7156	3	1	OG		None	None
MTCH1	Mitochondrial carrier 1	HGNC:17586	3	1	ODEG	House-keeping gene	None	None
MYC	MYC proto-oncogene, bHLH transcription factor	HGNC:7553	3	1	OG	Oncogen, Essential, Transcription factor	None	Literature
NDUFA12	NADH:ubiquinone oxidoreductase subunit A12	HGNC:23987	3	1	ODEG	House-keeping gene	None	None
NOTCH1	Notch receptor 1	HGNC:7881	2.7	1	OG	Tumor suppressor gene, Oncogen	DisGeNET, Malacards and Literature	Literature
NOTCH2	Notch receptor 2	HGNC:7882	2.7	1	ODEG	Tumor suppressor gene	Malacards and Literature	Literature
NOX1	NADPH oxidase 1	HGNC:7889	3	1	ODEG		None	None
NRAS	NRAS proto-oncogene, GTPase	HGNC:7989	2.7	2	OG	Oncogen	None	None
PGD	Phosphogluconate dehydrogenase	HGNC:8891	3	1	OG	Essential	None	None
PI4KA	Phosphatidylinositol 4-kinase alpha	HGNC:8983	3	1	ODEG	Essential	None	None
PICALM	Phosphatidylinositol binding clathrin assembly protein	HGNC:15514	3	1	ODEG	House-keeping gene	None	None
PKM	Pyruvate kinase M1/2	HGNC:9021	2.7	1	ODEG	Essential	Literature	Literature
PLCG1	Phospholipase C gamma 1	HGNC:9065	2.7	3	OG	House-keeping gene	None	None
PODXL	Podocalyxin like	HGNC:9171	3	1	OG		None	None
PPP1CA	Protein phosphatase 1 catalytic subunit alpha	HGNC:9281	2.7	1	ODEG	House-keeping gene, Tumor suppressor gene	None	None

Supplementary Table S4. (Continued)

Gene symbol	Approved name	HGNC ID	Significant score	Nº significant cluster present	Interactor type	Gene type	Evidence of gene-disease relationship	Evidence of gene-ENS relationship
PPP1R1B	Protein phosphatase 1 regulatory inhibitor subunit 1B	HGNC:9287	3	1	ODEG	Tumor suppressor gene	None	None
PPP1R9A	Protein phosphatase 1 regulatory subunit 9A	HGNC:14946	2.7	1	ODEG		None	None
PPT1	Palmitoyl-protein thioesterase 1	HGNC:9325	3	1	OG	House-keeping gene	None	None
PRKD1	Protein kinase D1	HGNC:9407	3	1	OG		None	None
PRPF40A	Pre-mRNA processing factor 40 homolog A	HGNC:16463	3	1	ODEG	House-keeping gene	None	None
PRPF8	Pre-mRNA processing factor 8	HGNC:17340	2.7	1	OG	House-keeping gene, Essential	None	None
PTEN	Phosphatase and tensin homolog	HGNC:9588	3	1	OG	House-keeping gene, Tumor suppressor gene	Literature	Literature
PTPN1	Protein tyrosine phosphatase non-receptor type 1	HGNC:9642	2.7	1	OG	House-keeping gene, Tumor suppressor gene	None	None
PTPN11	Protein tyrosine phosphatase non-receptor type 11	HGNC:9644	2.7	1	OG	House-keeping gene, Tumor suppressor gene, Oncogen	Literature	None
PTPN14	Protein tyrosine phosphatase non-receptor type 14	HGNC:9647	2.7	1	ODEG		None	None
PTPN6	Protein tyrosine phosphatase non-receptor type 6	HGNC:9658	2.7	2	ODEG	Tumor suppressor gene	Literature	None
PTPRF	Protein tyrosine phosphatase receptor type F	HGNC:9670	2.7	1	OG		None	None
PTPRJ	Protein tyrosine phosphatase receptor type J	HGNC:9673	3	1	OG	Tumor suppressor gene	None	None
RAB1A	RAB1A, member RAS oncogene family	HGNC:9758	3	1	ODEG	House-keeping gene	None	None
RAC2	Rac family small GTPase 2	HGNC:9802	2.7	2	ODEG		Literature	None
RAC3	Rac family small GTPase 3	HGNC:9803	3	1	OG		None	None
RASA1	RAS p21 protein activator 1	HGNC:9871	3	1	OG		None	None
RBMXL1	RBMX like 1	HGNC:25073	3	1	OG		None	None
RHOA	Ras homolog family member A	HGNC:667	3	1	OG	House-keeping gene, Tumor suppressor gene, Oncogen, Essential	HuGENet and Literature	Literature
RHOB	Ras homolog family member B	HGNC:668	3	1	ODEG	Tumor suppressor gene	Literature	None

Supplementary Table S4. (Continued)

Gene symbol	Approved name	HGNC ID	Significant score	Nº significant cluster present	Interactor type	Gene type	Evidence of gene-disease relationship	Evidence of gene-ENS relationship
RHOV	Ras homolog family member V	HGNC:18313	3	1	ODEG		None	None
ROBO2	Roundabout guidance receptor 2	HGNC:10250	3	1	ODEG		None	None
RPS6KA1	Ribosomal protein S6 kinase A1	HGNC:10430	3	1	OG		None	None
SCARB1	Scavenger receptor class B member 1	HGNC:1664	3	1	OG		None	None
SCRIB	Scribble planar cell polarity protein	HGNC:30377	3	1	OG	Tumor suppressor gene	None	None
SERPINH1	Serpin family H member 1	HGNC:1546	3	1	ODEG		None	None
SFN	Stratifin	HGNC:10773	3	1	OG	Tumor suppressor gene	None	None
SLC19A1	Solute carrier family 19 member 1	HGNC:10937	3	1	OG		None	None
SLC20A2	Solute carrier family 20 member 2	HGNC:10947	3	1	OG		None	None
SLC25A16	Solute carrier family 25 member 16	HGNC:10986	3	1	OG		None	None
SLC25A23	Solute carrier family 25 member 23	HGNC:19375	3	1	ODEG		None	None
SLC25A6	Solute carrier family 25 member 6	HGNC:10992	2.7	1	OG	House-keeping gene	None	None
SLC35B2	Solute carrier family 35 member B2	HGNC:16872	3	1	ODEG		None	None
SLC6A15	Solute carrier family 6 member 15	HGNC:13621	3	1	OG		None	None
SNAP23	Synaptosome associated protein 23	HGNC:11131	3	1	OG		None	None
SOX4	SRY-box transcription factor 4	HGNC:11200	3	1	OG	Oncogen, Transcription factor	DisGeNET and Literature	Literature
SRC	SRC proto-oncogene, non-receptor tyrosine kinase	HGNC:11283	2.7	1	OG	Oncogen	Literature	Literature
SREBF2	Sterol regulatory element binding transcription factor 2	HGNC:11290	3	1	OG	House-keeping gene, Transcription factor	None	None
STOM	Stomatin	HGNC:3383	3	1	OG		None	None
SUMO2	Small ubiquitin like modifier 2	HGNC:11125	3	1	OG	House-keeping gene	None	None
SURF4	Surfeit 4	HGNC:11476	3	1	ODEG	House-keeping gene	None	None
SYNCRIP	Synaptotagmin binding cytoplasmic RNA interacting protein	HGNC:16918	3	1	OG	House-keeping gene	None	None
TBC1D10A	TBC1 domain family member 10A	HGNC:23609	3	1	OG		None	None
TFRC	Transferrin receptor	HGNC:11763	3	2	OG	Essential	None	None
THRAP3	Thyroid hormone receptor associated protein 3	HGNC:22964	3	1	OG	House-keeping gene	None	None

Supplementary Table S4. (Continued)

Gene symbol	Approved name	HGNC ID	Significant score	Nº significant cluster present	Interactor type	Gene type	Evidence of gene-disease relationship	Evidence of gene-ENS relationship
TMEM164	Transmembrane protein 164	HGNC:26217	3	1	OG		None	None
TMEM87A	Transmembrane protein 87A	HGNC:24522	3	2	OG	House-keeping gene	None	None
TMTC4	Transmembrane O-mannosyltransferase targeting cadherins 4	HGNC:25904	3	1	OG		None	None
TRIP10	Thyroid hormone receptor interactor 10	HGNC:12304	3	1	ODEG		None	None
TULP3	TUB like protein 3	HGNC:12425	3	2	OG		None	None
TXNL4A	Thioredoxin like 4A	HGNC:30551	3	1	OG	Essential	None	None
UBE2I	Ubiquitin conjugating enzyme E2 I	HGNC:12485	3	1	OG	House-keeping gene, Essential	None	None
VCAM1	Vascular cell adhesion molecule 1	HGNC:12663	3	1	OG		None	Literature
XRCC3	X-ray repair cross complementing 3	HGNC:12830	3	2	OG	Essential	None	None
YAP1	Yes1 associated transcriptional regulator	HGNC:16262	2.7	1	ODEG	Tumor suppressor gene, Oncogen	Literature	None
YWHAE	Tyrosine 3-monooxygenase/tryptophan 5-monooxygenase activation protein epsilon	HGNC:12851	2.7	1	OG	House-keeping gene	Malacards and Literature	Literature
ZC3H18	Zinc finger CCCH-type containing 18	HGNC:25091	2.7	1	ODEG	House-keeping gene, Essential	None	None

ODEG, Other differential expressed gene in Hirschsprung; OG, Other gene; CTD, Comparative Toxicogenomics Database
