# Supplementary material for: Bioinformatics Prediction for Network-Based Integrative Multi-Omics Expression Data Analysis in Hirschsprung Disease
Source: Biomolecules. 2024 Jan 30;14(2):164. doi: 10.3390/biom14020164 (PMC10886964; doi:10.3390/biom14020164)
Supplement: Supplementary file 1 [file biomolecules-14-00164-s001.zip › biomolecules-2784092-supplementary/Supplementary_files/Table S5.pdf]

Supplementary Table S5. Hirschsprung miRNA-Target Interaction information collected from three different databases.

Database	Interactions	miRNA	Gene
DIANA	16118	210	5338
miRTarbase	7268	375	2567
miRecords	1303	97	926
Overall number of unique elements	22227	430	6763
