# Supplementary material for: Bioinformatics Prediction for Network-Based Integrative Multi-Omics Expression Data Analysis in Hirschsprung Disease
Source: Biomolecules. 2024 Jan 30;14(2):164. doi: 10.3390/biom14020164 (PMC10886964; doi:10.3390/biom14020164)
Supplement: Supplementary file 1 [file biomolecules-14-00164-s001.zip › biomolecules-2784092-supplementary/Supplementary_files/Table S6.pdf]

Supplementary Table S6. List of predicted Hirschsprung-related miRNAs

Ranking	miRNA	TRS[1]	Number of attachment to different databases	Target of De circRNA in HSCR	Target of HSCR-related lncRNA	Aberrant expression level in plasma	Aberrant expression level in colon	Evidence of mirna-disease relationship	HSCR Target genes
1	hsa-miR-1-3p	31.957	3		YES			None	ACKR3, ACTB, AKT3, ARHGEF7, ATP2A2, ATP2B1, B9D1, BANF1, BDNF, BLVRB, BMP4, BRCA1, BRIP1, BSG, CAV1, CBR1, CDC42, CDH2, CPNE8, CSE1L, CSGALNACT2, CSTF2T, CTSD, CX3CL1, DECR1, DEPDC1, DHCR7, DVL3, EDN1, EGFR, FANCC, FANCD2, FANCG, FANCI, FBXO7, FERMT2, FGF1, FHL1, FLOT1, FLOT2, FN1, FRS2, GEMIN2, GJA1, H3C11, H3C2, HAND2, HIRA, HNRNPU, HSPB1, IGF1, IL11, KRAS, L1CAM, LIMA1, LRP8, LZTFL1, MAPK3, MLLT11, MSX1, MYC, NCAM1, NOG, NOTCH2, NRAS, PAX3, PGD, PGRMC2, PHAX, PICALM, PLCG1, PODXL, PRKCI, RAB1A, RAC3, RAD51, RBP4, RFWD3, RHOB, ROCK1, SEMA3A, SHC1, SLC2A1, SLC6A15, SNAI2, SNAP23, SOX9, SRC, SREBF2, STMN3, STOM, SUMO2, SYT11, TFRC, TGFB2, TH, TMEM138, TMEM231, TMEM237, TMEM87A, ZNF609
2	hsa-miR-16-5p	20.57	3		YES			None	ACTB, ACTR2, AKT3, APP, ATP2A2, ATP2B1, AXIN2, B3GNT2, BACE1, BDNF, BMI1, BRCA1, CADM1, CDC42, CDC5L, DVL3, DYRK1A, FRS2, IKBKB, KDR, KIFAP3, KRAS, MBOAT7, PKM, PLAG1, PPT1, RAB1A, RAD51C, RASA1, SLC6A4, SLIT2, SON, UBE2T, WNT3A, YAP1
3	hsa-miR-15a-5p	9.499	3		YES			None	ACTB, AKT3, APP, ATP2A2, ATP2B1, BDNF, BMI1, BRCA1, CADM1, CDC42, DYRK1A, FRS2, KIFAP3, KLF4, PKM, RAD51C, RET, SLIT2, SON, WNT3A, YAP1
4	hsa-miR-29b-3p	9.072	3					None	AKT3, BACE1, BAG6, CDC42, CTNND1, DNMT1, DNMT3B, ESR1, FBN1, ITGB1, LIMA1, MYC, NID1, PIK3CG, PLAG1, PTEN, RORA, S100B, SCAF11, SERPINH1, TGFB2
5	hsa-miR-124-3p	8.023	3					None	ARRB2, BACE1, BDNF, CAV1, CDH2, CTNNB1, CTNND1, CYP3A5, DEPDC1, DHCR7, DNMT3B, DTNA, DVL2, ENO2, FERMT2, FLOT1, FLOT2, GEMIN5, ITGB1, JAG1, KIFAP3, KLF4, MAD2L2, MECP2, MLLT11, MYC, NID1, NRAS, PGRMC2, PHACTR4, PODXL, PRKD1, PRPF40A, RAD51, RFWD3, ROCK1, ROCK2, RPS6KA1, SEC24C, SERPINH1, SHC1, SNAI2, SNAP23, STOM, SURF4, TMEFF2, TUBA1A, YAP1
6	hsa-miR-17-5p	7.586	3					None	ACTB, ADAM9, APP, ATP2A2, BMP2, BRCA2, CELSR3, CEP120, DNMT1, EGR2, ETV1, FRS2, GAB1, HSPB2, MYC, PRKD1, PTEN, UBR4
7	hsa-miR-27a-3p	7.333	2					None	ATP2B1, BMI1, CDC42BPB, CTNNB1, CTNND1, DPYD, DYRK1A, EGFR, ELAVL4, FBN1, FZD3, GATA2, IGF1, KRAS, NOTCH2, PAX3, PIK3CG, PLAG1, PRKCSH, PRPF8, PTPRJ, RORA, RREB1, SEMA6A, SLC6A8, STOM, TMTC4, YAP1
8	hsa-let-7a-5p	6.934	3				YES	HMDD and Literature	ACTB, ATP2A2, BCOR, CHD7, CUL2, DDHD1, EDN1, EGFR, FBN1, FZD3, IGF2, KRAS, MYC, NRAS, PKM, PLAG1, PLAGL2, PRPF8, RHOB, WNT1

Supplementary Table S6. (Continued)

Ranking	miRNA	TRS[1]	Number of attachment to different databases	Target of De circRNA in HSCR	Target of HSCR-related lncRNA	Aberrant expression level in plasma	Aberrant expression level in colon	Evidence of mirna-disease relationship	HSCR Target genes
9	hsa-miR-221-3p	5.664	3					Literature	DVL2, ESR1, FERMT2, GJA1, GRB10, HOXB5, ICAM1, KIT, PTEN, RAB1A, RAD51, ZEB2
10	hsa-let-7b-5p	5.585	2					None	ACTB, ATP2A2, BCOR, CUL2, EDN1, FBN1, FZD3, IGF2, NRAS, PCDHAC2, PLAG1, PLAGL2, PRPF8, RFWD3, RHOB, SNAP23, YWHAE
11	hsa-miR-29c-3p	4.99	3				YES	Literature	AKT3, BACE1, BAG6, CDC42, CTNND1, DNMT3B, FBN1, ITGB1, KLF4, LIMA1, NRAS, PLAG1, PTEN, RORA, SCAF11, SERPINH1, SLC19A1
12	hsa-miR-20a-5p	4.926	2					None	ADAM9, AHNAK, APP, CEP120, DNMT1, EGR2, ETV1, FRS2, GJA1, KIT, LIMA1, MYC, NRG1, PTEN, RORA, SERPINF1, SOX4, UBR4
13	hsa-miR-181a-5p	4.849	3		YES	YES	YES	Literature	AHNAK, BRCA1, CDX2, CEP120, CTNNB1, DUSP6, ESR1, FRS2, HNRNPU, JMJD1C, KRAS, MAP2K1, NOTCH1, NOTCH2, NRAS, PLAG1, PLAU, PRDX3, PTEN, PTPN11, PTPN22, RORA, SON, TCF4, TMEM138, ZIC2
14	hsa-miR-29a-3p	4.724	3					None	AHNAK, AKT3, BACE1, BAG6, CDC42, CTNND1, DNMT1, DNMT3B, FBN1, IGF1, ITGB1, KLF4, LIMA1, MYC, PLAG1, PTEN, ROBO1, RORA, S100B, SCAF11, SERPINH1
15	hsa-miR-200b-3p	4.72	3			YES	YES	HMDD and Literature	BMI1, DNMT1, DNMT3B, FERMT2, FN1, HOXB5, IKBKB, KDR, NOTCH1, RHOA, ROCK2, SHC1, WNT1, ZEB2
16	hsa-miR-15b-5p	4.575	3		YES			None	ACTB, AKT3, ATP2A2, ATP2B1, AXIN2, BMI1, CDC42, DYRK1A, INSR, KDR, KIFAP3, PPT1, RAB1A, SLIT2, SON
17	hsa-miR-200c-3p	4.476	3				YES	HMDD and Literature	ATRX, BMI1, DNMT3B, EDNRA, FN1, GEMIN2, HOXB5, IKBKB, JAG1, KDR, KRAS, NCAM1, NOTCH1, NTF3, PTEN, RHOA, ROCK2, SHC1, TUBB3, ZEB2
18	hsa-miR-26a-5p	3.99	3					None	AHNAK, ARHGAP35, B3GNT2, DNMT3B, ESR1, IGF1, JAG1, MYC, NOS2, NRAS, PIK3CG, PLAG1, PRDX3, PTEN, PTPN1, RFWD3, RHOB, SMAD1, TFRC, YWHAE
19	hsa-let-7i-5p	3.962	3					None	ACTB, ASCL1, ATP2A2, BMP4, DDHD1, FANCD2, FBN1, FZD3, IGF1, IGF2, NRAS, PLAG1, PLAGL2, PRPF8
20	hsa-miR-34c-5p	3.362	3					Literature	MAP2K1, MAPT, MYC, NOTCH1, NOTCH2, NOTCH4, SOX2
21	hsa-miR-107	3.302	3				YES	HMDD and Literature	ACTB, APP, AXIN2, B3GNT2, BACE1, CAV1, CDC42, CTNNB1, KLF4, NOTCH2, PLAG1, RAD51, SALL4, SON, STOM, TUBA1A, UBE2T
22	hsa-miR-93-5p	3.283	2					None	ADAM9, AHNAK, APP, BCOR, CAV1, CELSR3, CEP120, DYRK1A, FOXA1, FRS2, PTEN, RORA, TRIP10, UBR4
23	hsa-miR-27b-3p	3.222	3					None	BMI1, CX3CL1, DPYD, DYRK1A, EDNRA, EGFR, FZD3, NOTCH1, PAX3, PAX7, PRKCSH, PRPF8, RET, RORA, RREB1, SEMA6A, SHC1, TMTC4
24	hsa-let-7g-5p	2.773	2					None	ACTB, ATP2A2, BMI1, DDHD1, FBN1, FN1, FZD3, IGF2, KRAS, MYC, NRAS, PLAG1, PLAGL2, PRPF8
25	hsa-miR-106b-5p	2.723	2					None	ADAM9, AHNAK, APP, CEP120, LIMA1, PTEN, RORA, UBR4

Supplementary Table S6. (Continued)

Ranking	miRNA	TRS[1]	Number of attachment to different databases	Target of De circRNA in HSCR	Target of HSCR-related lncRNA	Aberrant expression level in plasma	Aberrant expression level in colon	Evidence of mirna-disease relationship	HSCR Target genes
26	hsa-miR-424-5p	2.706	3		YES		YES	Literature	ACTB, AEBP2, AKT3, APP, ATP2B1, CDX2, CUL2, KDR, KIFAP3, MAP2K1, PLAG1, PTCH1, SLIT2, SON
27	hsa-miR-30a-5p	2.702	3					DisGeNet and Literature	AHNAK, ATP2A2, BCOR, BDNF, CUL2, DNMT1, FAF2, IFNGR2, NCAM1, NOTCH1, PLAGL2, RHOB, SMAD1, SOX4, TRAF3IP2
28	hsa-let-7f-5p	2.519	2					None	ACTB, ASS1, ATP2A2, DDHD1, FBN1, FZD3, IGF2, KIF1A, NRAS, PLAG1, PRPF8
29	hsa-miR-181b-5p	2.479	3		MEG3			None	AHNAK, BRCA1, CDX2, ESR1, HNRNPU, MAP2K1, PLAG1, PTEN, TMEM138
30	hsa-miR-30c-5p	2.429	3					None	AHNAK, ATP2A2, BCOR, CDC42, CUL2, DNMT1, FAF2, IFNGR2, IL11, NOTCH1, PLAGL2, RHOB, SMAD1, SNAI2, SOX4, UBE2I
31	hsa-miR-103a-3p	2.387	2					None	ACTB, APP, B3GNT2, CAV1, CTNNB1, CUL4A, DVL1, KLF4, PGD, PTEN, RAD51, SON, STOM, UBE2T
32	hsa-miR-30e-5p	2.264	3					None	AHNAK, BCOR, BMI1, CUL2, FAF2, IFNGR2, NOTCH1, PLAGL2, RHOB, SOX4, UBE2I
33	hsa-miR-222-3p	2.159	2				YES	Literature	ESR1, GJA1, GRB10, ICAM1, KIT, PTEN
34	hsa-miR-19a-3p	2.116	3		YES			None	AHNAK, AKT1, DNMT1, ESR1, KIT, MECP2, PTEN, RHOB, RORA
35	hsa-miR-23b-3p	1.967	3		YES			None	AHNAK, CHD7, CTNNB1, GJA1, HNRNPU, MARCKSL1, NOTCH1, NOTCH2, PLAU, PRDX3, PRKCSH, PTEN, SRC
36	hsa-miR-30d-5p	1.789	2					None	AHNAK, BCOR, CELSR3, CUL2, FAF2, FOXA1, IFNGR2, NOTCH1, PLAGL2, RHOB, SMAD1, SOX4
37	hsa-miR-92a-3p	1.695	2			YES		Literature	CTNNB1, DNMT1, DUSP6, FBN1, IL10RB, KLF4, KRAS, MYC, PTEN, RORA, RREB1, SOX4, SRC
38	hsa-miR-34b-3p	1.612	2					None	JAG1, MAP2K1, MYC, NOTCH1, NOTCH2, NOTCH4, SOX2
39	hsa-miR-125b-5p	1.472	3					None	AHNAK, AKT1, DUSP6, EGFR, ERBB2, ERBB3, GLI1, IGF2, NES, NTRK3, PODXL, PPP1CA, RPS6KA1, SMO, UBE2I
40	hsa-miR-30b-5p	1.427	2					None	AHNAK, ATP2A2, BCOR, CUL2, DNMT1, FAF2, IFNGR2, NOTCH1, PLAGL2, RHOB, SMAD1, SOX4
41	hsa-miR-106a-5p	1.235	3					None	ADAM9, APP, BMP2, CDX2, CEP120, ERCC1, PTEN, UBR4
42	hsa-miR-23a-3p	1.182	2		YES			None	AHNAK, CHD7, CTNNB1, FANCG, FOXA1, GJA1, MARCKSL1, PRDX3, PRKCSH, PTEN, PTPN11, TFRC
43	hsa-miR-34a-5p	1.171	3					Literature	AGTR1, AKT1, AXIN2, CDH2, ERBB2, FLOT2, GAB1, GAPDH, GFRA3, GORASP2, JAG1, KCNH2, KIT, KLF4, L1CAM, LIMA1, LZTFL1, MAD2L2, MAP2K1, MYC, NOTCH1, NOTCH2, PIK3CG, PRKD1, RAC2, RAD51, SOX2, SRC, SURF4, VCL, WNT1

Supplementary Table S6. (Continued)

Ranking	miRNA	TRS[1]	Number of attachment to different databases	Target of De circRNA in HSCR	Target of HSCR-related lncRNA	Aberrant expression level in plasma	Aberrant expression level in colon	Evidence of mirna-disease relationship	HSCR Target genes
44	hsa-miR-98-5p	1.163	2					None	ACTB, CTNND1, DDHD1, EDN1, FBN1, FZD3, IGF1, MYC, NRAS, PLAGL2, SALL4
45	hsa-miR-141-3p	1.06	3				YES	DisGeNet, HMDD and Literature	HOXB5, JAG1, PTEN, SHC1, TGFB2, YAP1, ZEB2
46	hsa-miR-155-5p	1.056	3		YES			None	ACTR2, AGTR1, CEP41, EDN1, ICAM1, KRAS, MECP2, MITF, MLLT11, MYC, PCDH9, PICALM, PODXL, PTEN, PTPRJ, RAD51, RHOA, RORA, SMAD1, SOX9, TCF4, YWHAE
47	hsa-let-7e-5p	1.038	3					None	ACTB, FZD3, IGF1, MARCKSL1, MYC, PLAGL2, WNT1
48	hsa-miR-21-5p	1.019	3					None	BMI1, CADM1, CUL2, EGFR, ERBB2, HNRNPU, ICAM1, JAG1, NTF3, PLAG1, PTEN, PTPN14, RASA1, RHOB, SERPINI1, SOX2, TGFB2
49	hsa-miR-20b-5p	0.736	3				YES	Literature	ADAM9, AKT3, BRCA1, CEP120, ESR1, PTEN, UBR4
50	hsa-miR-195-5p	0.717	2		YES		YES	CTD, DisGeNet, HMDD and Literature	ACTB, AKT3, ATP2A2, BACE1, CDC42, INSR, KDR, PKM, RET, SLIT2, SON, YAP1
51	hsa-miR-32-5p	0.717	2					None	CTNNB1, FBN1, IL10RB, KLF4, PTEN, RORA, SOX4, SRC
52	hsa-miR-200a-3p	0.704	3			YES	YES	HMDD and Literature	ATRX, CTNNB1, DNMT1, EGFR, GAPDH, GEMIN2, GJA1, HOXB5, MNX1, PTEN, SHC1, TGFB2, YAP1, ZEB2
53	hsa-miR-19b-3p	0.672	3		HOTTIP			None	DNMT1, ESR1, PTEN, RORA
54	hsa-miR-26b-5p	0.657	2					None	AHNAK, IGF1, JAG1, PTEN, SMAD1, YWHAE
55	hsa-miR-199a-5p	0.621	2					None	CAV1, CDH2, EDN1, ERBB2, ERBB3, IKBKB, JAG1, KRAS, TGFB2
56	hsa-miR-25-3p	0.597	2			YES		Literature	ATP2A2, CYP2B6, ERBB2, FANCI, HAND2, KLF4, KRAS, PTEN, RORA, SLX4, SOX4
57	hsa-miR-148a-3p	0.59	3				YES	Literature	DNMT1, DNMT3B, IKBKB, IRS1, PTPN14, ROCK1, SOX4, TGFB2, UBR4, WNT1
58	hsa-miR-130a-3p	0.566	3					None	CDC5L, ESR1, GJA1, KLF4, MECP2, MYC, OAS3, PTEN, ROCK1, SOX4, TAC1
59	hsa-let-7d-5p	0.539	1					None	ACTB, DEPDC1, EDN1, FBN1, PLAGL2, PTPN6, RHOB
60	hsa-miR-181c-5p	0.531	3		YES			None	BRCA1, CDX2, KRAS, MECP2, NOTCH4, PTEN, TMEM138
61	hsa-miR-145-5p	0.517	3		YES			None	ABCC1, AHNAK, BRAF, CDH2, CFTR, CTNND1, EGFR, ESR1, ILK, IRS1, KLF4, MYC, NRAS, PODXL, RASA1, ROBO2, ROCK1, RREB1, SOX2, SOX9, TGFB2
62	hsa-miR-22-3p	0.442	3		YES			None	ACTR2, AKT1, BDNF, BSG, CREBBP, ERBB2, ERBB3, ESR1, HDAC6, HTR2C, PTEN, RAB1A, SLC2A1, TFRC, UBR4, WNT1, YAP1

Supplementary Table S6. (Continued)

Ranking	miRNA	TRS[1]	Number of attachment to different databases	Target of De circRNA in HSCR	Target of HSCR-related lncRNA	Aberrant expression level in plasma	Aberrant expression level in colon	Evidence of mirna-disease relationship	HSCR Target genes
63	hsa-let-7c-5p	0.396	2					None	ACTB, MYC, NRAS, RHOB
64	hsa-miR-18a-5p	0.375	3			YES		Literature	ACTB, DNMT1, ESR1, PTEN, RORA
65	hsa-miR-125a-5p	0.358	3					None	AHNAK, AKT1, CD34, EDN1, EGFR, ERBB2, ERBB3, NTRK3, PIK3CG
66	hsa-miR-218-5p	0.345	2			YES	YES	CTD, DisGeNet, HMDD and Literature	BMI1, CDH2, EGFR, GJA1, IKBKB, KIT, MITF, RET, ROBO1, SMO
67	hsa-miR-148b-3p	0.274	3					None	DNMT1, DNMT3B, FLOT2, NOG, NRAS, ROBO1, ROCK1, SLC2A1, SOX4, WNT1
68	hsa-miR-92b-3p	0.274	2					None	FBN1, PTEN, SOX4, SRC, TCF4
69	hsa-miR-429	0.259	1				YES	HMDD and Literature	DNMT1, HOXB5, MYC, PTEN, SHC1, SOX2, ZEB2
70	hsa-miR-7-5p	0.23	3		YES			None	ABCC1, ACTB, DYRK1A, EGFR, HNRNPU, HOXB5, IRS1, KLF4, LEMD3, PAX6, PIK3CG, PRPF8, UBE2I, XRCC2
71	hsa-miR-128-3p	0.229	2				YES	CTD, DisGeNet and Literature	BMI1, DCX, DYRK1A, EGFR, EPHB2, IGF1, KLF4, NTRK3, PFKL, PTEN, RELN, RET, SNAI2, SREBF1, SREBF2, STOM
72	hsa-miR-101-3p	0.214	2					None	AEBP2, APP, CFTR, CTNNB1, MITF, MNX1, NOTCH1, RHOA, RORA, SOX9, SYNCRIP, ZEB2
73	hsa-miR-214-3p	0.159	2				YES	DisGeNet and Literature	CADM1, CTNNB1, JAG1, NRAS, POR, PTEN, SUFU, UBE2I
74	hsa-miR-126-3p	0.155	3					None	ADAM9, AKT1, CADM1, CXCR4, DNMT1, FAT3, IRS1, KRAS, PIK3CG, ROCK1, SOX2, TCF4, VCAM1
75	hsa-miR-100-5p	0.151	2					None	ACKR3, AKT1, B3GNT2, CD14, CEP104, EDN1, FKBP5, HSPB2, HSPE1, HYLS1, IGF2, MAP2, MMP10, ROCK1, SEMA3C, STOM, TMEFF2
76	hsa-miR-133b	0.138	2					None	AKT1, CDC42, CXCR4, EGFR, GLI1, GLI3, GSTP1, KCNH2, PKM, RHOA
77	hsa-miR-185-5p	0.138	2					None	AKT1, CDC42, DNMT1, EPHB2, MYC, NTRK3, RHOA, SCARB1, SREBF1, SREBF2
78	hsa-miR-203a-3p	0.138	2					None	BANF1, BMI1, CAV1, CUL1, EDNRA, MMP10, SNAI2, SRC, TCF4, ZEB2
79	hsa-miR-205-5p	0.138	2					None	ERBB2, ERBB3, EZR, PTEN, SMAD1, SRC, ZEB2
80	hsa-miR-338-3p	0.138	2	YES			YES	Literature	CDH2, PKM, SMO, SOX4, ZEB2
81	hsa-miR-301a-3p	0.129	2					None	OAS3, PTEN
82	hsa-miR-130b-3p	0.11	2					None	CDC5L, ERBB2, IGF1, ITGB1, PTEN
83	hsa-miR-497-5p	0.103	2		YES			None	ACTB, IKBKB, MAP2K1, SON
84	hsa-miR-301b-3p	0.085	2					None	OAS3, PTEN

Supplementary Table S6. (Continued)

Ranking	miRNA	TRS[1]	Number of attachment to different databases	Target of De circRNA in HSCR	Target of HSCR-related lncRNA	Aberrant expression level in plasma	Aberrant expression level in colon	Evidence of mirna-disease relationship	HSCR Target genes
85	hsa-miR-181d-5p	0.071	1		YES			None	AHNAK, BRCA1, KRAS, TMEM138
89	hsa-miR-326	0.069	2	YES	YES			None	FGF1, GLI1, NOTCH1, NOTCH2, PKM, SMO
86	hsa-miR-17-3p	0.069	2					None	ICAM1, KDR, PTEN, SON, SOX4
87	hsa-miR-224-5p	0.069	2					None	CDC42, CXCR4, EDNRA, KRAS, PRDX3, SLC25A16
88	hsa-miR-324-5p	0.069	2	YES				None	GLI1, SMO
90	hsa-miR-342-3p	0.069	2					None	DNMT1, SREBF1, SREBF2
91	hsa-miR-30e-3p	0.063	1					None	AHNAK, CAV1, NOTCH2, PLAG1, YWHAE
92	hsa-miR-24-3p	0.057	3				YES	DisGeNet, HMDD and Literature	AHNAK, BRCA1, MARCKSL1, MYC, NOTCH1, NOTCH2, PTPRF, SH2B1, SLC6A4, SON
93	hsa-miR-182-5p	0.052	3					None	ADCY6, BDNF, BRCA1, CADM1, CHL1, FLOT1, MITF, PTEN, SNAI2
94	hsa-miR-193b-3p	0.052	3					None	ESR1, KIT, KRAS, PLAU, RAD51
95	hsa-miR-204-5p	0.052	3					None	AHNAK, ATP2B1, BDNF, CDC42, CXCR4, DVL3, EZR, IL11, ITGB4, MAP2K1, SHC1, SNAI2, SOX4, TCF4
96	hsa-miR-9-5p	0.052	3				YES	Literature	ABCC1, AHNAK, BACE1, CD34, CDX2, CUL4A, CXCR4, FBN1, GDNF, NOTCH1, NOTCH2, NTRK3
97	hsa-miR-186-5p	0.05	2					None	AKAP12, DEPDC1, GJA1
98	hsa-miR-196a-5p	0.042	3					HuGeNet	IKBKB, ITGB1, MYC, NRP2, PRPF8
99	hsa-miR-320a	0.035	2		YES			None	ACTR2, BMI1, CTNNB1, KITLG, LYN, MYC, NOD2, PTEN, SYNCRIP, TFRC
100	hsa-miR-140-3p	0.034	1	YES				None	FN1, MARCKSL1
101	hsa-miR-193a-3p	0.034	2					None	ERBB2, KRAS, PLAU, PTEN, TGFB2
102	hsa-miR-194-5p	0.034	1				YES	Literature	BMI1, CDH2
103	hsa-miR-199b-5p	0.034	2					None	DYRK1A, ERBB2, GRB10, JAG1, KIT, PODXL
104	hsa-miR-202-3p	0.034	1					None	GLI1
105	hsa-miR-33a-5p	0.034	2				YES	Literature	ACTB, ADAM9, ARL3, MYC, SRC, SREBF1
106	hsa-miR-494-3p	0.034	2		YES	YES	YES	Literature	AHNAK, AKT1, BMI1, CFTR, CXCR4, EGFR, MAP2K1, MCC, MYC, PTEN, SYNCRIP
107	hsa-miR-10b-5p	0.028	3		YES		YES	Literature	AKT1, BCOR, KLF4, NOTCH1, PAX6, PTCH1, PTEN
108	hsa-miR-142-3p	0.028	2	YES			YES	HMDD and Literature	ATP2A2, CSE1L, DYRK1A, EGR2, FAM83D, LRP8, ROCK2, THBS4

Supplementary Table S6. (Continued)

Ranking	miRNA	TRS[1]	Number of attachment to different databases	Target of De circRNA in HSCR	Target of HSCR-related lncRNA	Aberrant expression level in plasma	Aberrant expression level in colon	Evidence of mirna-disease relationship	HSCR Target genes
109	hsa-miR-30a-3p	0.028	3				YES	DisGeNet and Literature	AHNAK, MECP2, NOTCH1, NOTCH2, TUBA1A
110	hsa-miR-138-5p	0.019	3					None	AKT1, FERMT2, RELN, ROCK2, S100A1, SNAI2, SOX4, SOX9, YAP1, ZEB2
111	hsa-miR-1826	0.017	1					None	CTNNB1, MAP2K1
112	hsa-miR-20a-3p	0.017	1					None	EGR2, PTEN, SMO
113	hsa-miR-211-5p	0.017	1					Malacards	IL11, MAP2K1, SOX4
114	hsa-miR-296-3p	0.017	1				YES	Literature	ICAM1
115	hsa-miR-448	0.017	1					None	MAP2K1
116	hsa-miR-449a	0.017	1					None	FLOT2, MAP2K1, MYC, NOTCH1
117	hsa-miR-506-3p	0.017	1					None	CDH2, DNMT3B, FLOT1, GLI3, ROCK1, SNAI2, YAP1
118	hsa-miR-509-3-5p	0.017	1					None	PODXL
119	hsa-miR-675-3p	0.017	1					None	SMAD1
120	hsa-miR-320b	0.016	2		YES			None	ACTR2, MYC, NOD2
122	hsa-miR-320c	0.013	2		YES			None	ACTR2, NOD2
121	hsa-miR-196b-5p	0.013	2					None	AKT1, ITGB1, MYC, PIK3CG, PRPF8
123	hsa-miR-374a-5p	0.013	1					None	ADAM9, CTNNB1, SOX4, TCF4
124	hsa-miR-423-5p	0.011	1					None	DVL3, HNRNPU
125	hsa-miR-199a-3p	0.009	2			YES	YES	Literature	FN1, IGF1, KDR, YAP1
126	hsa-miR-320d	0.008	1		YES			None	ACTR2
127	hsa-miR-10a-5p	0.006	2		YES			None	BCOR, BDNF, CHL1, NOD2, PIK3CG, PTEN, RORA
128	hsa-miR-548c-5p	0.006	1					None	RORA
129	hsa-miR-548j-5p	0.006	1					None	RORA
130	hsa-miR-199b-3p	0.005	1					None	FN1
131	hsa-miR-34b-5p	0.005	3					None	AXIN2, CTNNB1, MYC
132	hsa-miR-625-5p	0.005	3					None	FRS2, ILK, NTRK3, SOX2
133	hsa-miR-153-3p	0.003	2					None	PTEN, SNAI2, ZEB2

Supplementary Table S6. (Continued)

Ranking	miRNA	TRS[1]	Number of attachment to different databases	Target of De circRNA in HSCR	Target of HSCR-related lncRNA	Aberrant expression level in plasma	Aberrant expression level in colon	Evidence of mirna-disease relationship	HSCR Target genes
134	hsa-miR-449c-5p	0.003	2					None	MYC
135	hsa-miR-944	0.003	1	YES				None	AEBP2, CTNNB1
136	hsa-miR-548h-3p	0.002	1					None	MMGT1
137	hsa-miR-548z	0.002	1					None	MMGT1

[1]TSR, Total relevance score.
