# Supplementary material for: Bioinformatics Prediction for Network-Based Integrative Multi-Omics Expression Data Analysis in Hirschsprung Disease
Source: Biomolecules. 2024 Jan 30;14(2):164. doi: 10.3390/biom14020164 (PMC10886964; doi:10.3390/biom14020164)
Supplement: Supplementary file 1 [file biomolecules-14-00164-s001.zip › biomolecules-2784092-supplementary/Supplementary_files/Table S7.pdf]

Supplementary Table S7. Dysregulated CircRNAs identified in Hirschsprung's disease with predicted Target miRNAs proposed in this study.

ID	CircRNAs	Reference	Predicted Target miRNAs
1	hsa_circ_0000615	[87]	hsa-miR-142-3p
			hsa-miR-324-5p
			hsa-miR-338-3p
2	hsa_circ_0001313	[86]	hsa-miR-338-3p
3	hsa_circ_0002069	[88]	hsa-miR-338-3p
4	hsa_circ_0007502	[88]	hsa-miR-140-3p
			hsa-miR-142-3p
			hsa-miR-324-5p
			hsa-miR-326
			hsa-miR-338-3p
			hsa-miR-944
5	hsa_circ_0007646	[88]	hsa-miR-324-5p
6	hsa_circ_0007704	[88]	hsa-miR-142-3p
7	hsa_circ_0019744	[88]	hsa-miR-338-3p
8	hsa_circ_0021098	[88]	hsa-miR-324-5p
			hsa-miR-326
			hsa-miR-338-3p
9	hsa_circ_0021104	[88]	hsa-miR-324-5p
			hsa-miR-326
			hsa-miR-338-3p
10	hsa_circ_0038689	[88]	hsa-miR-142-3p
			hsa-miR-338-3p
11	hsa_circ_0059926	[89]	hsa-miR-140-3p
12	hsa_circ_0059963	[88]	hsa-miR-326
13	hsa_circ_0072255	[88]	hsa-miR-324-5p
			hsa-miR-326
14	hsa_circ_0077582	[88]	hsa-miR-324-5p
15	hsa_circ_0081648	[88]	hsa-miR-140-3p
			hsa-miR-142-3p
			hsa-miR-324-5p
			hsa-miR-326
			hsa-miR-338-3p
			hsa-miR-944
16	hsa_circ_0087023	[88]	hsa-miR-326
