# Supplementary material for: Bioinformatics Prediction for Network-Based Integrative Multi-Omics Expression Data Analysis in Hirschsprung Disease
Source: Biomolecules. 2024 Jan 30;14(2):164. doi: 10.3390/biom14020164 (PMC10886964; doi:10.3390/biom14020164)
Supplement: Supplementary file 1 [file biomolecules-14-00164-s001.zip › biomolecules-2784092-supplementary/Supplementary_files/Table S8.pdf]

Supplementary Table S8. Hirschsprung related lncRNAs with predicted Target miRNAs proposed in this study.

lncRNA	Predicted Target miRNAs
AFAP1-AS1	hsa-mir-155-5p
HOTTIP	hsa-mir-19a-3p; hsa-mir-19b-3p
LINC01518	hsa-mir-1-3p
MEG3	hsa-mir-10a-5p; hsa-mir-10b-5p; hsa-mir-145-5p; hsa-mir-15a-5p; hsa-mir-15b-5p; hsa-mir-16-5p; hsa-mir-81a-5p; hsa-mir-181b-5p; hsa-mir-181c-5p; hsa-mir-181d-5p; hsa-mir-195-5p; hsa-mir-22-3p; hsa-mir-23a-3p; hsa-mir-23b-3p; hsa-mir-320a; hsa-mir-320b; hsa-mir-320c; hsa-mir-320d; hsa-mir-326; hsa-mir-424-5p; hsa-mir-494-3p; hsa-mir-497-5p; hsa-mir-7-5p
