# Supplementary material for: Bioinformatics Prediction for Network-Based Integrative Multi-Omics Expression Data Analysis in Hirschsprung Disease
Source: Biomolecules. 2024 Jan 30;14(2):164. doi: 10.3390/biom14020164 (PMC10886964; doi:10.3390/biom14020164)
Supplement: Supplementary file 1 [file biomolecules-14-00164-s001.zip › biomolecules-2784092-supplementary/Supplementary_files/Table S9.pdf]

Supplementary Table S9. Most significant results (until Top 5) obtained for categories: Chromosomal location (miRBase), Expressed in tissue (Tissue Atlas), Cell-type specific (Atlas), ExRNA forms (miRandola) and Gender and Age, after ORA analysis by miEAA performed on predicted HSCR-related miRNAs.

Category	Subcategory	Enrichment	P-adjusted	Expected	Observed
Chromosomal location (miRBase)	Chromosome 13	over-represented	2.24×10^{-3}	3	11
	Chromosome 9	over-represented	1.42×10^{-2}	6	15
	Chromosome 1	over-represented	3.73×10^{-2}	12	21
Expressed in tissue (Tissue Atlas)	Thyroid	over-represented	2.14×10^{-10}	88	119
	Epididymis	over-represented	3.47×10^{-8}	86	114
	Colon	over-represented	3.94×10^{-8}	87	114
	Lung	over-represented	5.33×10^{-8}	86	113
	Skin	over-represented	4.58×10^{-7}	90	114
	Lymph node	over-represented	3.30×10^{-2}	86	96
Cell-type specific (Atlas)	Neuronal stem cell	over-represented	4.06×10^{-2}	1	5
ExRNA forms (miRandola)	Ago2	over-represented	3.58×10^{-12}	29	60
	Exosome	over-represented	3.58×10^{-12}	89	117
	Microvesicle	over-represented	9.16×10^{-12}	20	47
	Circulating	over-represented	1.13×10^{-10}	91	116
	Microparticle	over-represented	7.67×10^{-3}	2	5
Gender and Age	Negatively correlated with age	over-represented	1.86×10^{-6}	20	39
	Upregulated in male	over-represented	1.86×10^{-3}	11	21
	Positively correlated with age	under-represented	5.57×10^{-3}	39	28
	Upregulated in female	under-represented	2.12×10^{-2}	16	9
	Age dependent	over-represented	2.36×10^{-2}	60	67
